# An Explanatory Model of Violent Behavior, Self-Concept, and Alcohol, Tobacco, and Cannabis Consumption in Secondary Education Students

**DOI:** 10.1155/2023/1971858

**Published:** 2023-04-15

**Authors:** Eduardo Melguizo-Ibáñez, Gabriel González-Valero, Georgian Badicu, Filipe Manuel Clemente, Ana Filipa Silva, Pilar Puertas-Molero

**Affiliations:** ^1^Department of Didactics of Musical, Plastic and Corporal Expression, Faculty of Education Sciences, University of Granada, 18071 Granada, Spain; ^2^Department of Physical Education and Special Motricity, Faculty of Physical Education and Mountain Sports, Transilvania University of Brasov, 500068 Brasov, Romania; ^3^Escola Superior Desporto e Lazer, Instituto Politécnico de Viana do Castelo, Rua Escola Industrial e Comercial de Nun'Álvares, 4900-347 Viana do Castelo, Portugal; ^4^Instituto de Telecomunicações, Delegação da Covilhã, 1049-001 Lisboa, Portugal; ^5^Research Center in Sports Performance, Recreation, Innovation and Technology (SPRINT), 4960-320 Melgaço, Portugal

## Abstract

**Background:**

Current scientific evidence establishes that regular physical activity engagement provides numerous physical and mental benefits. Thus, the present research aims at examining the relationships between violent behavior, self-concept, and consumption of alcohol, tobacco, and cannabis. Specifically, two objectives were defined: (a) analyze and establish the relationships between violent behaviors, the different dimensions of self-concept, and the consumption of alcohol, tobacco, and cannabis as a function of physical activity engagement, (b) define and examine a proposed explanatory model, and (c) analyze the effect of self-concept on alcohol and tobacco consumption and physical activity engagement based on the explanatory model developed.

**Methods:**

For this purpose, a nonexperimental (ex post facto), descriptive, and cross-sectional study was conducted. For data collection, a sociodemographic questionnaire was administered alongside the Self-Concept Form 5 and the School Victimization Scale.

**Results:**

It was shown that individuals engaging in more than three hours of physical exercise per week scored more highly on the social, family, physical, and emotional aspects of self-concept, whilst those who do not meet this criterion scored more highly on the academic aspect and on physical and verbal victimization.

**Conclusions:**

The present research concludes that engagement in more than 3 hours of physical activity per week led to benefits in a number of domains of self-concept whilst, at the same time, increasing levels of violence.

## 1. Introduction

Adolescence is a stage of human development recognized as a transition period between childhood and adulthood [1] in which individuals seek integration into the adult world [2]. This crucial period of change is characterized by physical development and changes at psychological and social levels [3].

During this stage of human development, violent behaviors tend to increase as peers seek to demonstrate superiority [4]. In relation to aggression and violence, different theories have been put forward that attempt to explain causal etiological factors [4]. Biological theories explain aggressive behavior based on biological variables associated with hormonal factors, such as testosterone, and deficiencies in neurotransmitters, such as serotonin [5]. Likewise, theoretical learning models establish that aggressive behaviors have their origin in classical conditioning, with the linking of certain stimuli leading to aggression [5]. Research by Chen et al. [6] indicates that the most common manifestations among adolescents are verbal (insults, threats, and putdowns), followed by relational aggressions (social isolation) and physical aggressions. Despite this, Vannucci et al. [7] point to the virtual environment as causing an increase in violent acts, mainly verbal in nature, through social networks [8].

In this sense, most research focuses on the study of aggressors and victims ([9]); however, there is a growing line of interest that focuses on examining the characteristics of victims [10]. A study carried out by Benítez-Sillero et al. [11] concluded that adolescents mainly fell foul of this type of behavior because of their physical appearance. This negatively impacted their self-esteem and the mental image they had of themselves.

It has been shown that people who suffer bullying behavior or harassment show a lower level of self-concept [12]. Self-concept is defined as a mental representation held by individuals of themselves when interacting with the environment around them [13]. This term is derived from a unidimensional conception and has scientifically evolved to assume a multidimensional perspective. Initially, Shavelson et al. [14] proposed self-concept to be a multidimensional construct composed of five dimensions: academic, emotional, social, physical, and family. The development of the different areas of self-concept takes place throughout life [15]. Based on the above, an uneven development of the areas of self-concept has been observed during adolescence [16]. Research carried out by Melguizo-Ibáñez et al. [17] affirms that adolescents are more invested in the social aspect of the construct, with this aspect taking on greater importance and relevance than the family setting. Due to the importance given to the social area, some young people start consuming harmful substances, mainly alcohol and tobacco, as a means to reaffirm their image with respect to their peers [18]. In addition, the early onset of substance use can lead to the use of other harmful substances like cannabis, which have been shown to have a particularly negative impact on the health of adolescent girls [19]. The consumption of this type of substance has been positively related with a worse health status in young people [20]. In this case, regular consumption of alcoholic beverages, together with tobacco use, produces more than three million deaths annually [20], whilst regular cannabis use has been related to an increased risk of serious mental problems including depression, anxiety, and personality disorders [21].

Similarly, another problem that typically arises during adolescence pertains to reduced physical activity engagement [22]. This is due to an increased interest in more sedentary activities [23]. Regular engagement in any type of physical sports activity has been observed to produce physical and psychological improvements [24]. In this case, a study by Fraguela-Vale et al. [24] found that leading an active lifestyle produced improvements in the different areas of self-concept. Those engaging in physical activity or sports had higher physical self-concept, with the frequency of engagement being a particularly important element. Additionally, physical activity engagement has also been shown to be associated with a decrease in violent behaviors [25] as it helps to promote impulse control and stop violent behavior from originating. Research conducted by Benítez-Sillero et al. [9] established that individuals who suffered violent behavior at the hands of their peers were less likely to engage in physical activity and more likely to have a high body mass index and, as a result, have a lower state of fitness. According to findings reported by Archimi and Kuntsche [26], the latter is a factor behind young people starting to consume harmful substances at an early age. Likewise, a study conducted by the Health Department [27] concluded that higher levels of sedentary behavior were associated with a higher consumption of alcoholic beverages, tobacco, and cannabis.

Previous research has addressed the aforementioned variables independently [28]. The present study will be the first to develop and present a structural equation model that analyzes the effect of the variables described above as a function of the time adolescents spend engaging in physical activity.

The present study addresses the following research hypotheses:

**Hypothesis 1 .**

Participants who engage in more than 3 hours of physical activity per week will report a negative link between harmful substance use and self-concept.

**Hypothesis 2 .**

Young people who meet the established physical sports criterion will reflect a negative relationship between harmful substance use and violent behavior.

**Hypothesis 3 .**

Participants who engage in more than 3 hours of physical exercise per week will exhibit a positive relationship between substance use and self-concept.

**Hypothesis 4 .**

Young people who do not meet the established physical sports criterion will exhibit a positive relationship between substance use and violent behavior.

Finally, specific research objectives were defined to explore the relationships between violent behaviors, self-concept, and the consumption of harmful substances. Specifically, (a) analyze and establish the relationships between violent behaviors, the different dimensions of self-concept, and the consumption of alcohol, tobacco, and cannabis as a function of physical activity engagement, (b) define and examine a proposed explanatory model, and (c) analyze the effect of self-concept on alcohol and tobacco consumption and physical activity engagement based on the explanatory model developed.

## 2. Material and Methods

### 2.1. Design and Participants

A descriptive, comparative, cross-sectional, and nonexperimental (ex post facto) research study was conducted with adolescents from different high schools in the province of Granada (Spain). In terms of the context and characteristics of the sample, it belongs to the eastern part of the province of Granada. Likewise, this area shows a medium-low socioeconomic level. In addition, all the participants belong to the compulsory secondary education stage. The sample consisted of a total of 706 adolescents (13.92 ± 1.31), of which 56.1% were female (*n* = 396) and 43.9% were male (*n* = 310). In terms of distribution according to school year, 33.7% (*n* = 238) were undertaking their first year, 24.6% (*n* = 174) their second year, 27.2% (*n* = 192) their third year, and 14.4% (*n* = 102) their fourth and final year of compulsory secondary education. The socioeconomic status of the sample was also homogeneous as data were collected from different areas of the province of Granada. Data collection was carried out following receipt of informed consent from the minors' legal guardians. Parents received a letter from the schools informing them about the study aims. Sampling error was 2.57% with 95% confidence.

### 2.2. Instruments

#### 2.2.1. Sociodemographic Questionnaire

This questionnaire was used to collect sociodemographic data such as participants' gender and age. In addition, it gathered information on physical activity engagement. In this case, the dichotomous question proposed by Arufe et al. [29] was used to examine whether participants were physically active (do you engage in more than 3 hours of physical activity outside of school hours?), with a yes or no response being possible. The State Survey on Drug Use in Secondary Education [27] was used to assess substance use frequency. This instrument was designed by the Spanish government delegation for the National Plan on Drugs (PNSD).

#### 2.2.2. Self-Concept Questionnaire Form 5

This instrument was developed by García and Musitu [30]. It consists of 30 items that are measured on a 5-point Likert scale, where 1 is “never” and 5 is “always”. Self-concept is grouped into the five dimensions of emotional self-concept, physical self-concept, social self-concept, and academic self-concept. The questionnaire produced a reliability index of *α* = 0.831.

#### 2.2.3. School Victimization Scale

The scale was developed by Mynard and Joseph [31]. The present research used the Spanish version adapted by Cava et al. [32]. This scale is composed of 20 items responded to on a Likert scale (1=never; 4=always), which assesses three types of victimization, namely, relational victimization, physical victimization, and verbal victimization. With regard to the internal consistency of the questionnaire, a score of *α* = 0.911 was produced.

### 2.3. Procedure

The first step was to conduct a literature review in order to contextualize the issue and identify reliable instruments for data collection. In this case, the literature review was carried out in the Web of Science and Scopus databases. This was also used to study the instruments that showed the highest degree of reliability and to focus this study. Likewise, the time range of this review was delimited from 2015 to 2022. Subsequently, the Department of Didactics of Musical, Artistic and Corporal Expression within the Faculty of Education Sciences drafted an information pack which was sent to participating high schools. This pack provided information about the study objectives and scope. Following this, schools forwarded a letter to the pupils' legal guardians. This letter informed parents about the research objectives and requested informed consent to enable the minors to participate in the study. Following receipt of informed consent, participants completed all the questionnaires via the Google Forms platform. This approach was necessary as researchers were unable to access the center due to the COVID-19 pandemic. Teachers at the centers were given instructions on how to resolve any potential doubts. Data were collected between January and February 2021. In order to avoid random responses, two questions were duplicated. This led to the elimination of 16 questionnaires which had not been properly completed. The study followed the principles established in the Helsinki Declaration of 1975 and was approved and supervised by the ethics committee at the University of Granada (1230/CEIH/2020).

### 2.4. Data Analysis

IBM Statistical Package for Social Sciences Amos 26.0 (IBM Corp, Armonk, NY, USA) was used to develop structural equation models. Such models enable relationships within adolescents who meet previously established physical sports engagement criteria to be compared with those who do not. All models are made up of eleven endogenous and three exogenous variables. With regard to the endogenous variables, causal relationships were examined in terms of the observed associations between indicators and the degree of measurement reliability. The above enables the error produced by the measurement of observed variables to be considered within the model. Two significance levels were proposed. The first set significance was at 0.05 level, whilst the second set significance was at 0.001.

**Figure 1:**
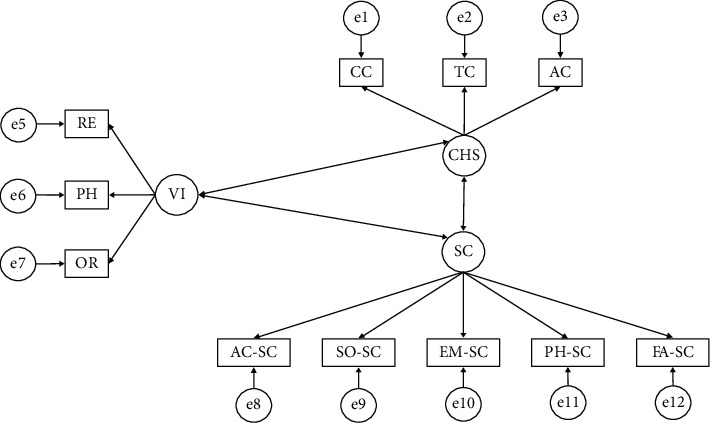
Proposed theoretical model. Note: self-concept (SC); academic self-concept (AC-SC); social self-concept (SO-SC); emotional self-concept (EM-SC); physical self-concept (PH-SC); family self-concept (FA-SC); violent behavior (VI); relational violence (RE); physical violence (PH); verbal violence (OR); consumption of harmful substances (CHS); cannabis consumption (CC); tobacco consumption (TC); alcohol consumption (AC).

In order to assess model fit, guidelines established by Maydeu-Olivares [33] and Kyriazos [34] were followed. According to these authors, goodness of fit should be assessed according to the Chi-squared, with nonsignificant *p* values indicating good model fit. To achieve good model fit, fit index values (CFI, GFI, and IFI) should be higher than 0.900. Finally, root mean square approximation (RMSEA) values should be below 0.100.  Figure 1 shows the proposed theoretical model.

## 3. Results

**Figure 2:**
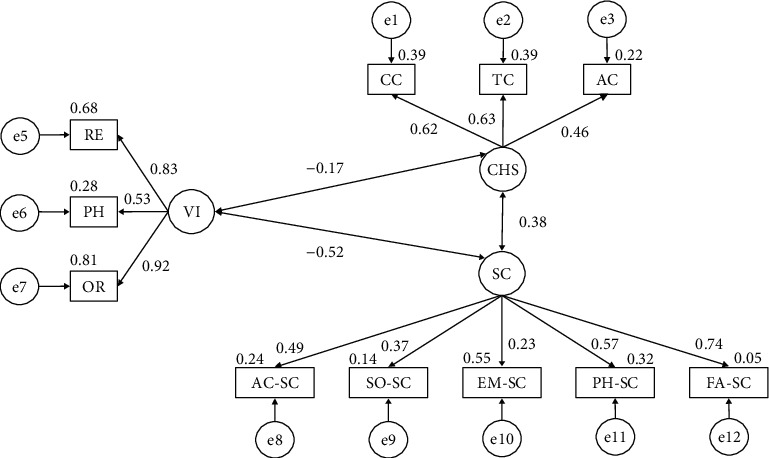
Model developed for the overall sample.

The model developed for the overall sample (Figure 2) obtained a nonsignificant *p* value (*X*^2^ = 251.630; df = 41; pl = 0.000); nevertheless, data cannot be interpreted independently due to sample size, influence, and susceptibility [33, 35]. For this reason, other standardized indices were used. The CFI was 0.878, the NFI was 0.859, the RFI was 0.811, the IFI was 0.879, and the TLI was 0.836, whilst the RMSEA was 0.085.

Table 1 shows the results obtained for the whole sample. A positive association was found between self-concept and its emotional (*r* = 0.229), physical (*r* = 0.569), family (*r* = 0.743), social (*r* = 0.375), and academic (*r* = 0.490) domains. Positive relationships were also observed between the use of harmful substances and alcohol (*r* = 0.621), tobacco (*r* = 0.627), and cannabis (*r* = 0.464) consumption. With regards to violence, positive relationships were observed with oral (*r* = 0.917), physical (*r* = 0.528), and relational (*r* = 0.827) behaviors. A negative relationship was also observed between violent behavior and substance use (*r* = −0.168) and self-concept (*r* = −0.517). Finally, a positive relationship was observed between substance use and self-concept (*r* = 0.378).

**Figure 3:**
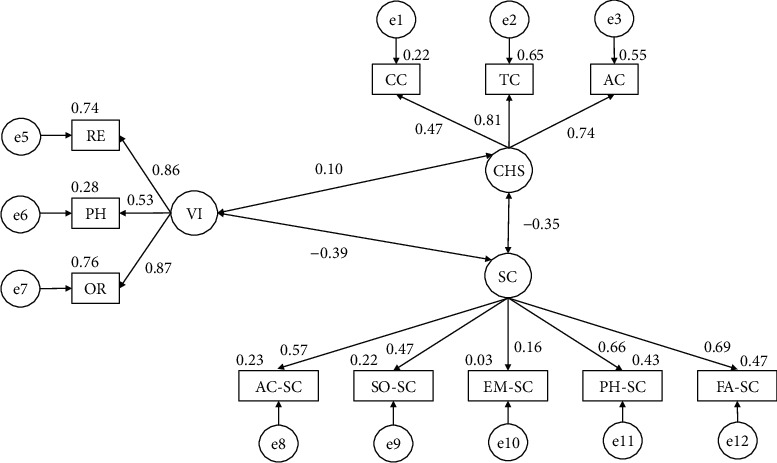
Model developed for participants who engage in more than 3 hours of PA per week.

The model developed for participants claiming to engage in more than one hundred and eighty minutes of physical activity per week (Figure 3) produced a nonsignificant *p* value (*X*^2^ = 144.320; df = 41; pl = 0.000). All fit indices were higher than 0.850, whilst the RMSEA was 0.073.

Table 2 and Figure 3 refer to the results of physically active participants. Harmful substance use was positively related with alcohol (*r* = 0.741), tobacco (*r* = 0.806), and cannabis (*r* = 0.468) consumption. Self-concept correlated positively with the emotional (*r* = 0.164), physical (*r* = 0.658), family (*r* = 0.687), social (*r* = 0.470), and academic (*r* = 0.574) domains of self-concept. Moving on to consider violent behaviors, such conduct was positively related with verbal (*r* = 0.873), physical (*r* = 0.529), and relational (*r* = 0.860) violence. Furthermore, self-concept was negatively correlated with substance use (*r* = −0.345) and disruptive behaviors (*r* = −0.391). Finally, violent behavior was positively related with substance use (*r* = −0.095).

**Figure 4:**
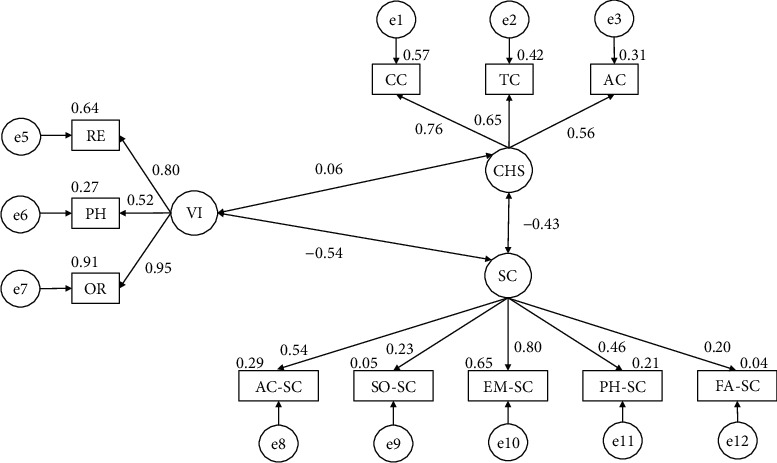
Model developed for participants who do not engage in more than 3 hours of PA per week.

The model developed from data pertaining to participants who did not meet the established physical activity sports engagement criteria (Figure 4) produced a nonsignificant *p* value (*X*^2^ = 230.264; df = 41; pl = 0.000). In this case, fit indices were all higher than 0.850, whilst the RMSEA was 0.072.

Table 3 shows the results obtained for participants who do not meet the physical criteria. It shows that the consumption of harmful substances is positively related with alcohol (*r* = 0.756), tobacco (*r* = 0.650), and cannabis (*r* = 0.561) use. Further, with regards to self-concept, positive relationships were observed with the emotional (*r* = 0.202), physical (*r* = 0.463), family (*r* = 0.803), social (*r* = 0.229), and academic (*r* = 0.541) domains. In contrast, negative relationships were found between self-concept and substance use (*r* = −0.428) and violent behavior (*r* = −0.538). Finally, with regards to disruptive behaviors, positive links were observed with verbal (*r* = 0.953), physical (*r* = 0.519), and relational (*r* = 0.802) violence, in addition to with substance use (*r* = 0.059).

## 4. Discussion

The following discussion aims at comparing the present findings with those reported in other previously published research papers.

With regard to the model developed for the overall sample, a positive association was observed between the consumption of harmful substances and the multidimensional construct of self-concept. Similar outcomes have been reported by Chaput-Langlois et al. [36] who concluded that the consumption of harmful substances within an adolescent population had a positive impact on different adolescent outcomes. This being said, Melguizo-Ibáñez et al. [37] concluded that regular consumption of harmful substances had a negative impact on the academic, family, and physical domains of self-concept, due to the fact that it led to more negative perceptions of these aspects. With regard to the relationship between violent behaviors and self-concept, a negative relationship was observed between both variables. Similar findings were reported by Shemesh and Heiman [10] who highlighted that those individuals on the receiving end of such behavior had a more negative self-perception. Likewise, a study carried out by Shemesh and Heiman [10] suggested that this relationship may vary depending on whether an individual is the victim or perpetrator of such behaviors. Specifically, when adolescents engage in violent behaviors towards their peers they experience a boost in different domains of self-concept, with the social domain being particularly heightened [10]. Turning attention to the relationship between the consumption of harmful substances and violent behavior, a negative relationship was observed. Hugely contrasting outcomes were obtained by Willoughby et al. [38] who reported that the regular consumption of alcoholic beverages encourages these behaviors due to the state of drunkenness caused by such substances. With regard to tobacco consumption, a study conducted by Chau et al. [39] concluded that smoking did not encourage violent behavior, with the same also being found with regard to cannabis consumption.

Turning attention to the structural equation models developed, stronger relationships were observed in participants who did not engage in more than 3 hours of physical activity per week when it comes to alcohol and cannabis use, whilst relationships pertaining to tobacco use were stronger in those who were physically active. Castro-Sánchez et al. [40] found that adolescents who led an active lifestyle were less likely to use harmful substances. In contrast, Salvador-Pérez et al. [41] stated that, throughout adolescence, there is an increase in the use of harmful substances used by young people, especially when the use of such substances was initiated within the peer group.

Obtained outcomes pertaining to self-concept revealed stronger associations with the physical, social, and academic domains of self-concept in participants who were physically active. These findings coincide with those reported by Zurita-Ortega et al. [16]. Being physically active helps to improve the individual's physical self-image [42] whilst also helping them to fit in better with their peer groups [43]. Further, an association with better academic performance has been found due to the secretion of neurotransmitters [44]. In contrast, with regard to the emotional and family domains of self-concept, a stronger association was observed in participants who did not exceed 3 hours of physical activity per week. Very different results were obtained by Ubago-Jiménez et al. [44] and Yuce and Muz [45]. These studies [44, 45] conclude that the practice of regular physical activity together with mental work practices helps to reduce anxiety, depression, and stress levels.

With regard to violent behaviors, a stronger relationship was observed with oral violence in participants who did not engage in more than 3 hours of physical activity. These findings coincide with those reported by Peláez-Barrios and Vernetta-Santana [46], who reported that physically inactive adolescents tended to receive negative comments about their fitness and physical condition. Further, a stronger association between physical and relational violence was found in physically active participants in the present study. These outcomes coincide with those obtained by Castro-Sánchez et al. [40] who concluded that physical and relational violence were the most common types of violence employed by adolescents to discriminate against their peers. Nonetheless, research conducted by Song et al. [47] revealed a new trend, with relational violence emerging as the most commonly employed by adolescents.

With regard to the relationship between violent behavior and substance use, a stronger association was observed in physically active participants. In view of these results, May et al. [48] argue that the use of harmful substances may have an impact on increasing violent behavior due to the dependence provoked by these substances. Finally, in the present study, a negative relationship was observed between self-concept and substance use, as well as with negative behaviors. Highly contrasting outcomes were obtained by Usan and Salavera [49] who stated that alcohol and tobacco consumption, together with aggressive behavior, helped to improve social self-concept in adolescents.

## 5. Limitations and Future Perspectives

The present study is characterized by a number of limitations which are outlined below. The cross-sectional nature of the study means it is not possible to establish cause-effect relationships between variables over a longitudinal time period, and conclusions can only be applied to the studied time period. Likewise, findings should be considered carefully due to the nature of the study. Another limitation is that it is not possible to make any causal conclusions or determine the directionality of associations. The recruited sample belongs to a very specific geographic region meaning that findings cannot be generalized to a wider population. It should also be noted that variables such as family socioeconomic status and parental educational level were not considered in the present study.

With regard to future perspectives, a longitudinal study is being planned, in which the causal relationships between variables examined in the present investigation can be studied as a function of physical activity engagement. Finally, it would also be of interest to repeat the present study and incorporate additional variable to enable the influence of family socioeconomic background to be considered.

## 6. Conclusions

The model developed for the general population showed a positive relationship between self-concept and substance use, whilst a negative relationship was revealed between self-concept and violent behaviors and between substance use and disruptive behaviors.

Structural equation models showed differences in the association between variables as a function of weekly physical activity engagement. In the case of both proposed models, a negative relationship was observed between the consumption of harmful substances (alcohol, tobacco, and cannabis) and self-concept, with this association being stronger in individuals who did not meet the physical activity sports engagement criterion. Negative associations between self-concept and violent behaviors were also observed in both models, although a stronger relationship was observed in those young people who did not engage in more than 3 hours of physical activity per week. In addition, a positive relationship was observed between substance use and violent behavior, with the association being stronger in participants who engage in more than 3 hours of physical activity per week.

Finally, the present research showed that engagement in more than 3 hours of physical activity per week led to benefits in a number of domains of self-concept whilst, at the same time, increasing levels of violence. In view of these findings, the educational sphere is of vital importance given that positive and effective physical education classes are capable of reducing the levels of aggression in the physical activity and sports settings.

## Figures and Tables

**Table 1:** Outcomes for the proposed model for the overall sample.

Association	RW				SRW
	Estimation	SE	CR	*p*	Estimation
EM-SC ←SC	1.000				0.229
PH-SC ←SC	2.835	0.570	4.975	^∗∗∗^	0.569
FA-SC ←SC	3.375	0.665	5.076	^∗∗∗^	0.743
SO-SC ←SC	1.478	0.324	4.559	^∗∗∗^	0.375
AC-SC ← SC	2.344	0.483	4.857	^∗∗∗^	0.490
AC ← CHS	1.000				0.621
TC ← CHS	0.960	0.121	7.916	^∗∗∗^	0.627
CC ← CHS	0.370	0.048	7.698	^∗∗∗^	0.464
OR ← VI	1.000				0.917
PH ←VI	0.359	0.026	13.794	^∗∗∗^	0.528
RE ←VI	0.881	0.043	20.405	^∗∗∗^	0.827
SC ← → CHS	0.020	0.005	3.930	^∗∗∗^	0.378
VI ← → CHS	-0.027	0.009	-3.180	^∗∗^	-0.168
VI ← → SC	-0.047	0.010	-4.686	^∗∗∗^	-0.517

Note: ^∗∗^*p* ≤ 0.05 and ^∗∗∗^*p* ≤ 0.001.

**Table 2:** Outcomes of the proposed model for participants engaging in more than 3 hours of PA.

Association	RW				SRW
	Estimation	SE	CR	*p*	Estimation
AC←CHS	1.000				0.741
TC ←CHS	1.202	0.146	8.234	^∗∗∗^	0.806
CC ←CHS	0.232	0.032	7.201	^∗∗∗^	0.468
EM-SC ←SC	1.000				0.164
PH-SC ←SC	4.573	1.788	2.557	^∗∗^	0.658
FA-SC ←SC	4.215	1.645	2.562	^∗∗^	0.687
SO ←SC	2.558	1.027	2.491	^∗∗^	0.470
AC ←SC	4.165	1.642	2.536	^∗∗^	0.574
OR ←VI	1.000				0.873
PH ←VI	0.367	0.039	9.480	^∗∗∗^	0.529
RE ←VI	0.915	0.070	13.140	^∗∗∗^	0.860
VI ← → CHS	0.047	0.033	1.416	0.157	0.095
CHS ← → SC	-0.043	0.019	-2.247	^∗∗^	-0.345
VI ←→ SC	-0.022	0.009	-2.355	^∗∗^	-0.391

Note: ^∗∗^*p* ≤ 0.05 and ^∗∗∗^*p* ≤ 0.001.

**Table 3:** Outcomes of the proposed model for participants engaging in less than 3 hours of PA.

Association	RW				SRW
	Estimation	SE	CR	*p*	Estimation
AC←CHS	1.000				0.756
TC ←CHS	1.020	0.124	8.217	^∗∗∗^	0.650
CC ←CHS	0.467	0.060	7.814	^∗∗∗^	0.561
EM-SC ←SC	1.000				0.202
PH-SC ←SC	2.396	0.753	3.184	^∗∗^	0.463
FA-SC ←SC	4.323	1.298	3.331	^∗∗∗^	0.803
SO ←SC	1.037	0.402	2.581	^∗∗^	0.229
AC ←SC	2.770	0.850	3.258	^∗∗^	0.541
OR ←VI	1.000				0.953
PH ←VI	0.349	0.035	9.883	^∗∗∗^	0.519
RE ←VI	0.842	0.056	15.037	^∗∗∗^	0.802
VI ← → CHS	0.034	0.037	0.919	0.358	0.059
CHS ← → SC	-0.070	0.024	-2.897	^∗∗^	-0.428
VI ←→ SC	-0.047	0.015	-3.131	^∗∗^	-0.538

Note: ^∗∗^*p* ≤ 0.05 and ^∗∗∗^*p* < 0.001.

## Data Availability

The data used to support the findings of current study are available from the corresponding author upon request.
